# The Regulation of Catecholamine Biosynthesis by the Gas Transmitters Carbon Monoxide and Hydrogen Sulfide

**DOI:** 10.3390/cimb47090725

**Published:** 2025-09-05

**Authors:** Robert Dingley, Cameron Hourtovenko, James Lee, Sujeenthar Tharmalingam, T. C. Tai

**Affiliations:** 1 Medical Sciences Division, Northern Ontario School of Medicine University, Sudbury, ON P3E 2C6, Canada; rdingley@laurentian.ca (R.D.); chourtovenko1@laurentian.ca (C.H.); jlee9@laurentian.ca (J.L.); sutharmalingam@nosm.ca (S.T.); 2 School of Natural Sciences, Laurentian University, Sudbury, ON P3E 2C6, Canada; 3 Health Sciences North Research Institute, Sudbury, ON P3E 2H2, Canada

**Keywords:** gas transmitter, catecholamine, carbon monoxide, hydrogen sulfide, epinephrine, phenylethanolamine N-methyltranserase, hypertension, transcriptional regulation

## Abstract

The gas transmitters nitric oxide (NO), carbon monoxide (CO), and hydrogen sulfide (H_2_S) play important roles in physiological regulation, including adrenal function. Among them, only NO has been directly implicated in controlling catecholamine biosynthesis. This study examined whether CO and H_2_S exert similar effects by treating PC12 cells with a CO donor (CORM-2) or an H_2_S donor (NaHS), with or without glucocorticoid stimulation. Gene expression of tyrosine hydroxylase (*Th*), dopamine β-hydroxylase (*Dbh*), and phenylethanolamine N-methyltransferase (*Pnmt*) was assessed by RT-qPCR, and catecholamine release was measured by ELISA. We found that exogenous CO decreased *Th* and *Dbh* expression, attenuated glucocorticoid-induced upregulation of catecholamine biosynthesis genes, and differentially modulated dopamine and norepinephrine release. In contrast, exogenous H_2_S treatment had no significant effect. These findings identify CO as a novel regulator of catecholamine biosynthesis and highlight important differences among gas transmitters in stress-related signaling.

## 1. Introduction

Catecholamines are organic molecules that function as hormones and neurotransmitters and are vital to the physiological stress response, particularly the ‘fight or flight’ reaction, which is partly mediated through the hypothalamic–pituitary–adrenal (HPA) axis [1,2,3]. HPA axis activation induces a rapid increase in the biosynthesis and secretion of glucocorticoids (GCs) in the adrenal cortex, mainly cortisol, in humans. The adrenal medulla is exposed to very high concentrations of glucocorticoids [4]. GCs directly increase the release of catecholamines from the adrenal medulla [5] and increase both enzyme activity and expression of catecholamine biosynthesis genes [6].

The catecholamines dopamine, norepinephrine, and epinephrine are all produced in the same sequential enzymatic pathway [7]. Catecholamines, particularly epinephrine, are implicated in the development of essential hypertension. Increased plasma catecholamines have been linked with individuals who have essential hypertension [8]. The gene expression, protein levels, and activity of phenylethanolamine N-methyltransferase (PNMT), the enzyme responsible for epinephrine production, are increased in hypertensive rats compared to normotensive rats [9,10]. Additionally, several genetic linkage studies have indicated *Pnmt* as a potential candidate gene for hypertension in rats and humans [11,12,13]. The difference in *Pnmt* expression among hypertensive individuals is not due to polymorphisms, but likely due to altered gene regulation [14].

Gas transmitters, also known as gasotransmitters, are a class of gases that act as cellular transmitters [15]. Currently, the gases nitric oxide (NO), carbon monoxide (CO) and hydrogen sulfide (H_2_S) are widely considered the canonical gas transmitters [15]. These gas transmitters have all been shown to regulate blood pressure and are implicated in the development of hypertension. NO was initially identified as an endothelium-derived relaxing factor due to its ability to signal smooth muscle cells to vasodilate after being produced in endothelial cells, marking the beginning of gas transmitter research [16,17]. Decreased bioavailability of NO in the endothelium and specific polymorphisms of the gene responsible for endothelial NO production have been associated with hypertension [18,19]. CO has similarly been shown to support vasodilation. Polymorphisms in the promoter region of heme oxygenase 1 (HO-1) have been linked to the occurrence of cardiovascular disease [20]. Induction of HO in the spontaneously hypertensive rat (SHR), either by treatment with hemin [21] or by the introduction of human HO-1 expression vectors, reduces blood pressure [22]. The inhalation of CO by mice attenuated the development of angiotensin II-dependent hypertension [23]. The endogenous production of H_2_S is primarily through cysteine metabolism, which is catalyzed by several enzymes, but primarily cystathionine γ-lyase (CSE), cystathionine β-synthase (CBS), and 3-mercaptopyruvate sulfurtransferase (3-MST) [24]. H_2_S exhibits similar vasodilative activity. Lower circulating levels of H_2_S are correlated with higher blood pressure and could be a marker of hypertension [25]. SHRs have lower expression levels of H_2_S-producing enzymes compared to normotensive rats, and lowering H_2_S-producing enzyme activity leads to increased blood pressure in normotensive rats [26]. H_2_S predominantly induces vasodilation by modulating the activity of K^+^ and Ca^2+^ channels [25]. These studies highlight the potential for interactions between catecholamines and gas transmitters, particularly in the regulation of blood pressure and the development of hypertension.

All three of these gases have been shown to participate in regulating the HPA axis [27]. However, the effect of gas transmitters on catecholamine biosynthesis remains unclear. A previous study showed that treatment of cells derived from a pheochromocytoma of the rat adrenal medulla (PC12) with the NO donor sodium nitroprusside (SNP) results in increased expression of catecholamine biosynthesis genes alone and a potentiation in the responses of the genes to the synthetic GC, dexamethasone (DEX) [28]. However, the ability of CO and H_2_S to modulate the expression of catecholamine biosynthetic genes and any interactions with glucocorticoid signaling is not yet known. While HOs are expressed in adrenal tissue and have been implicated in adrenal steroidogenesis [29], the effect of endogenous CO on catecholamine biosynthesis has not been studied. The closest comparable work is an investigation of the reaction of carotid bodies’ catecholamine content to extremely high acute concentrations of CO that are more consistent with hypoxic conditions or carbon monoxide poisoning [30,31]. The activity of H_2_S-producing enzymes plays a vital role in maintaining adrenal cortex mitochondrial function and glucocorticoid production [32]. In rat adrenal chromaffin cells, H_2_S induces catecholamine release by inhibiting membrane depolarization [33]. In calf chromaffin cells, exogenous H_2_S donation increases Ca^2+^ levels, triggering exocytosis [34]. While H_2_S plays a significant role in adrenal function, its effects on catecholamine production are unknown.

In this study, the role of CO and H_2_S in regulating catecholamine production was investigated. PC12 cells were treated with the gas donors tricarbonyldichlororuthenium (II) dimer, also known as carbon monoxide-releasing molecule 2 (CORM-2), and sodium hydrosulfide (NaHS) to deliver CO and H_2_S, respectively. Changes in the expression of catecholamine biosynthetic genes tyrosine hydroxylase (*Th*), dopamine β-hydroxylase (*Dbh*), and phenylethanolamine N-methyltransferase (*Pnmt*) were measured via reverse transcription quantitative polymerase chain reaction (RT-qPCR), and overall production and release of catecholamines were measured with an enzyme-linked immunosorbent assay (ELISA).

## 2. Materials and Methods

### 2.1. Cell Culture

PC12 cells (Dr. Sushil Mahata, Department of Medicine, University of California, San Diego, CA USA) were maintained in Dulbecco’s modified Eagle’s medium (DMEM (Medi-Res Corp., Sudbury, ON, Canada) with 5% bovine calf serum (BCS; Hyclone Laboratories, Logan, UT, USA), 5% equine serum (ES; Hyclone), and 0.5 mL gentamycin sulfate supplements (50 mg/mL; EMD Chemicals, Gibbstown, NJ, USA). Cells were cultured at 37 °C with 5% CO_2_ in 100 mm TC-treated cell culture dishes (Medi-Res) in a humidified incubator (Thermo-Fisher Scientific, Hampton, NH, USA). Cells were grown to 80–90% confluency before being passed or used in an experiment. Prior to experimentations, cells were washed twice with PBS, trypsinized, and transferred to DMEM-containing charcoal-treated serum. For RNA extraction and ELISA experiments, cells were seeded in 6-well plates (Medi-Res) at a 1.5 × 10^6^ cells/well from a suspension of 5 × 10^5^ cells/mL, with 3 mL added per well. Following seeding, cells were allowed to adhere to plates for 20–24 h prior to beginning experiments.

### 2.2. Experimental Design

To determine the effect of CO on the expression of genes associated with catecholamine biosynthesis, PC12 cells were treated with the CO donor CORM-2 at concentrations of 10, 20, 50, 80 or 100 μM for 6 h. To determine the effect of H_2_S on the expression of genes associated with catecholamine biosynthesis, PC12 cells were treated with the H_2_S donor NaHS at concentrations of 20, 50, 80, 100 or 300 μM for 6 h. mRNA expression of key catecholamine biosynthetic genes was assessed by RT-qPCR across three replicates. A preliminary investigation was conducted to determine the effects of CO and H_2_S on catecholamine production and release. PC12 cells were treated with either 100 μM CORM-2 or 300 μM NaHS for 6 or 24 h. Following treatment, cell culture media were collected and catecholamine levels quantified using the 3-Cat ELISA across two replicates.

To determine the effect of CO and H_2_S on glucocorticoid-regulated expression of catecholamine biosynthesis genes, PC12 cells were pretreated with 100 μM CORM-2 or 300 μM NaHS for 15 min, followed by a 6 h treatment with or without 1 μM dexamethasone (DEX). mRNA levels of catecholamine biosynthetic genes were quantified via RT-qPCR across three replicates. A preliminary investigation was conducted to determine the effect of CO and H_2_S on the glucocorticoid-mediated catecholamine levels in culture media. PC12 cells were pretreated with either 100 μM CORM-2 or 300 μM NaHS, followed by a treatment with 1 μM DEX for either 6 or 24 h. Cell culture media was then collected, and catecholamine levels were measured using a 3-Cat ELISA across two replicates.

### 2.3. RT-qPCR

RNA was isolated using the TRIzol reagent (Sigma-Aldrich Corp., MO, USA); 6 h after drug treatment, total RNA was extracted following the manufacturer’s protocols. Cell culture media was aspirated, 500 μL TRIzol was added to each well; plates were frozen at −80 °C for 24 h. RNA was separated from the total cell extract using centrifugation after the addition of chloroform (Thermo-Fisher Scientific). The aqueous phase was separated, and RNA was precipitated using isopropanol. After washing with 70% ethanol, the RNA pellet was resuspended in 20 μL DEPC-treated water. RNA concentrations were determined using the Nanodrop ND-1000 spectrophotometer (Nanodrop Technologies, Inc., Wilmington, DE, USA), RNA concentrations were measured in duplicate using 1 μL of resuspended RNA. RNA was then treated with DNase1 per the manufacturer’s instructions (Sigma-Aldrich) to a final concentration of 2 μg of RNA/22 μL H_2_O and subsequently reverse transcribed using Mu-MLV reverse transcriptase (Promega, Madison, WI, USA) to result in a final concentration of 0.04 μg cDNA/μL H_2_O. PCR was carried out using the GoTaq Flexi DNA polymerase (Promega) with 1 x GoTaq^®^ master mix buffer (Promega), 2 ng/μL of forward and reverse primers, 100 ng cDNA sample, and water. PCR products were separated on 2% agarose gel and imaged by ChemiDoc Imagining System (BioRad Laboratories, Hercules, CA, USA). qPCR was performed in a 96-well plate using SYBR green master mix (Medi-Res), 2 ng/μL of forward and reverse primers and 30 ng sample cDNA and run in a QuantStudio Real-Time PCR System machine (Thermo-Fisher Scientific) using Design & Analysis 2 software (version 2.8.0). The RT-qPCR cycling parameters consisted of 95 °C for one minute, followed by a two-step denaturation and extension cycle of 95 °C for 15 s and 58–60 °C for 30 s for a total of 40 cycles. Plate reading was performed at the end of the extension phase. A DNA melt curve was conducted at the end of each RT-qPCR experiment to evaluate the specificity of amplification. The RT-qPCR data were analyzed using the 2^−∆∆Ct^ analysis method [35]. Gene expression was normalized to glyceraldehyde 3-phosphate dehydrogenase (*Gapdh*) and Ribosomal Protein L32 (*Rpl32*). Genes of interest were *Th*, *Dbh*, and *Pnmt*. Primer sequences are outlined in Table 1.

### 2.4. Catecholamine ELISA

To determine the catecholamine content produced and released by PC12 cells, culture media was collected after treatments. To prevent catecholamine degradation, EDTA (final concentration 10 mM) and sodium metabisulfite (final concentration 4 mM) were added to the media and frozen at −80 °C until analysis. Catecholamine content was measured via a 3-Catecholamine ELISA kit (Labor Diagnostika Nord (LDN), Nordhorn, Germany) following the manufacturer’s protocol. In brief, dopamine, norepinephrine, and epinephrine were extracted using a cis-diol-specific affinity gel, which was acylated and enzymatically converted. The solution was then aliquoted to individual microtiter strips for each catecholamine, and a competitive enzyme-linked immunosorbent assay (ELISA) was performed as per manufacturer’s instructions; absorbance at 450 nm was measured in Synergy HTX Multi-Mode Microplate Reader (Agilent Technologies, Inc., Santa Clara, CA, USA).

### 2.5. Data Analysis

qPCR data were generated from at least three independent experiments per assay, while ELISA data were generated from at least two independent experiments per assay. For both, technical replicates were averaged to obtain one value per biological replicate, and results are presented as mean ± SEM. Statistical analyses were performed using Jamovi software (version 2.3.28). Group differences were assessed using one-way ANOVA, and when significant effects were detected, post hoc Tukey’s multiple comparisons test was applied. Data were inspected for normal distribution and homogeneity of variance prior to analysis.

## 3. Results

### 3.1. Effects of CO on the Expression of Catecholamine Biosynthesis Genes

**Figure 1:**
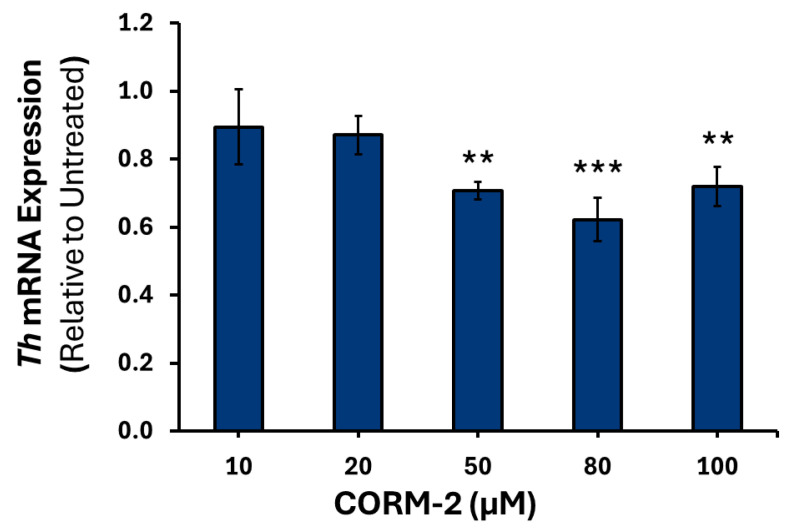
*Th* mRNA expression in response to varying CORM-2 treatments. PC12 cells were treated with either 10, 20, 50, 80 or 100 μM of CORM-2 (*n* = 3) for 6 h. Data are presented as the average fold change ±SEM of *Th* expression normalized to *Gapdh* and *Rpl32* and relative to an untreated control across three replicates ** *p* < 0.01, *** *p* < 0.001).

**Figure 2:**
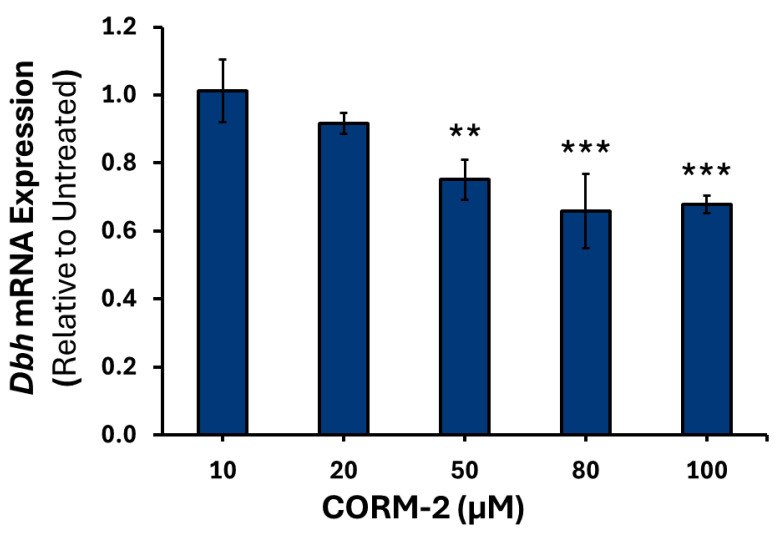
*Dbh* mRNA expression in response to varying CORM-2 treatments. PC12 cells were treated with either 10, 20, 50, 80 or 100 μM of CORM-2 (*n* = 3) for 6 h. Data are presented as the average fold change ±SEM of *Dbh* expression normalized to *Gapdh* and *Rpl32* and relative to an untreated control across three replicates ** *p* < 0.01, *** *p* < 0.001).

**Figure 3:**
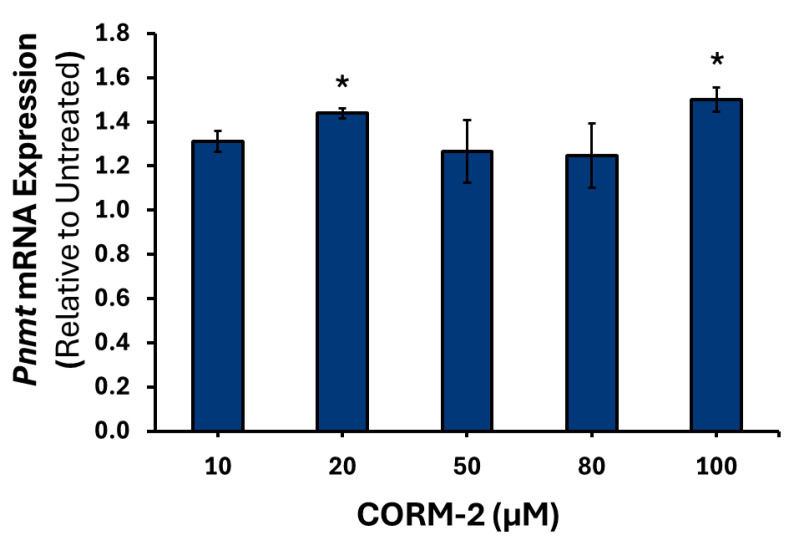
*Pnmt* mRNA expression in response to varying CORM-2 treatments. PC12 cells were treated with either 10, 20, 50, 80 or 100 μM of CORM-2 (*n* = 3) for 6 h. Data are presented as the average fold change ±SEM of *Pnmt* intronless expression normalized to *Gapdh* and *Rpl32* and relative to an untreated control across three replicates (* *p* < 0.05).

To confirm that the results observed were due to CO, treatment with inactive CORM-2 (iCORM-2) was performed (data not shown). Higher concentrations of CORM-2 induced moderate downregulation of *Th* mRNA, with 50 μM causing a 0.70-fold reduction (*p* = 0.001), 80 μM inducing a 1.61-fold decrease (*p* < 0.001), and 100 μM causing a 1.40-fold decrease (*p* < 0.01) relative to control (Figure 1). A similar pattern was observed for *Dbh* mRNA, with 50 μM causing a 1.33-fold decrease (*p* < 0.01), 80 μM a 1.53-fold decrease (*p* < 0.001), and 100 μM a 1.49-fold decrease (*p* < 0.001) compared to the control (Figure 2). However, a moderate increase in intronless *Pnmt* mRNA was detected at 20 μM, of 1.43-fold (*p* < 0.05) and 100 μM, of 1.50-fold (*p* < 0.05) (Figure 3).

### 3.2. Effects of H_2_S on the Expression of Catecholamine Biosynthesis Genes

**Figure 4:**
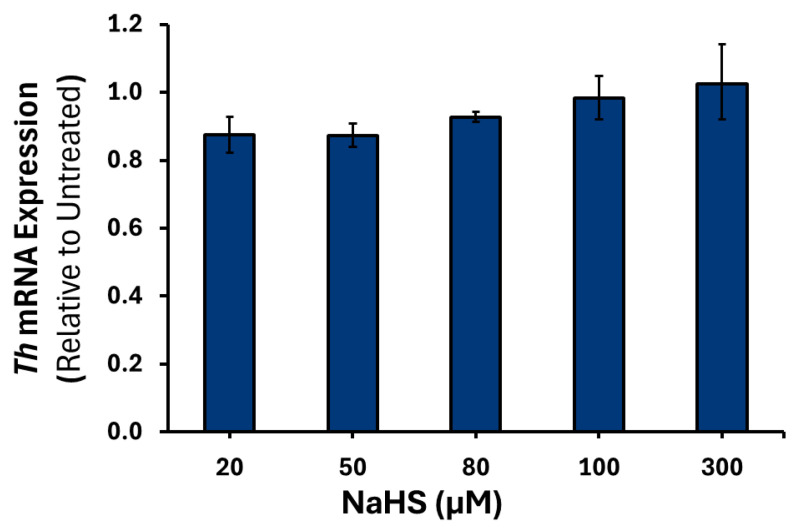
*Th* mRNA expression in response to varying NaHS treatments. PC12 cells were treated with either 20, 50, 80, 100 or 300 μM of NaHS (*n* = 3) for 6 h. Data are presented as the average fold change ±SEM of *Th* expression normalized to *Gapdh* and *Rpl32* and relative to an untreated control across three replicates.

**Figure 5:**
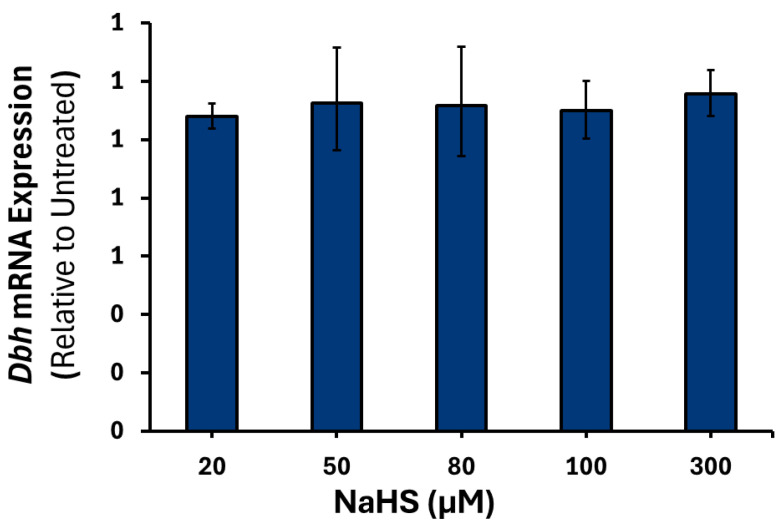
*Dbh* mRNA expression in response to varying NaHS treatments. PC12 cells were treated with either 20, 50, 80, 100 or 300 μM of NaHS (*n* = 3) for 6 h. Data are presented as the average fold change ±SEM of *Dbh* expression normalized to *Gapdh* and *Rpl32* and relative to an untreated control across three replicates.

**Figure 6:**
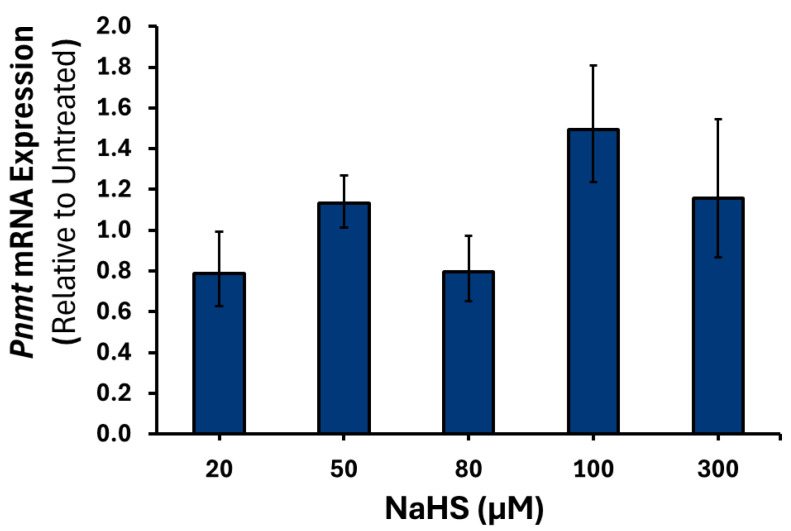
*Pnmt* mRNA expression in response to varying NaHS treatments. PC12 cells were treated with either 20, 50, 80, 100 or 300 μM of NaHS (*n* = 3) for 6 h. Data are presented as the average fold change ±SEM of *Pnmt* intronless expression normalized to *Gapdh* and *Rpl32* and relative to an untreated control across three replicates.

To confirm that the results observed were due to H_2_S, treatment with spent NaHS was performed (data not shown). No significant changes were observed in the expression of *Th*, *Dbh*, or *Pnmt* mRNA at any of the NaHS concentrations examined (Figure 4, Figure 5 and Figure 6).

### 3.3. Effects of CO and H_2_S on the Production and Release of Catecholamines

**Figure 7:**
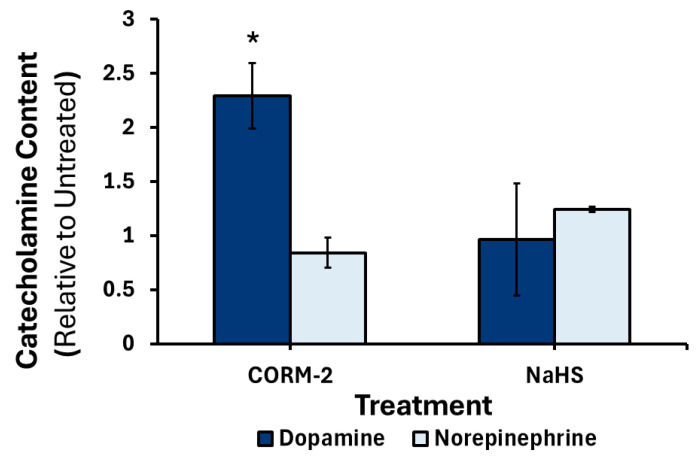
Changes in production and release of dopamine and norepinephrine in response to 24 h CORM-2 or NaHS treatment. PC12 cells were treated with 100 μM CORM-2 or 300 μM NaHS (*n* = 2) for 24 h. Data are presented as the average concentration ±SEM of dopamine or norepinephrine relative to an untreated control across two replicates (* *p* < 0.05).

Six hours after treatment with CORM-2 or NaHS there was no significant change in dopamine or norepinephrine content compared to control (data not shown). After 24 h, NaHS treatment resulted in no change in catecholamine content while CORM-2 treatment resulted in a 2.2-fold (*p* < 0.05) increase in dopamine levels compared to control (Figure 7).

### 3.4. Effects of CO and H_2_S on the Regulation of DEX-Associated Expression of Catecholamine Biosynthesis Genes

**Figure 8:**
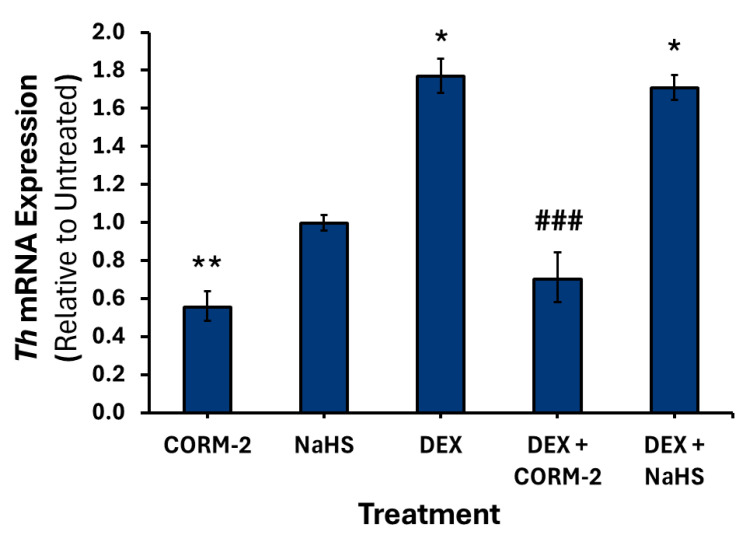
*Th* mRNA expression following dexamethasone and gas donor treatments. PC12 cells were pretreated with either 100 μM CORM-2 or 300 μM NaHS for 15 min, followed by treatment with or without 1 μM DEX (*n* = 3) for 6 h. Data are presented as the average fold change ±SEM of *Th* expression normalized to *Gapdh* and *Rpl32* and relative to an untreated control across three replicates (* *p* < 0.05, ** *p* < 0.01, ### *p* < 0.001). * Indicates a significant difference from untreated control, # represents a significant difference from DEX treatment.

**Figure 9:**
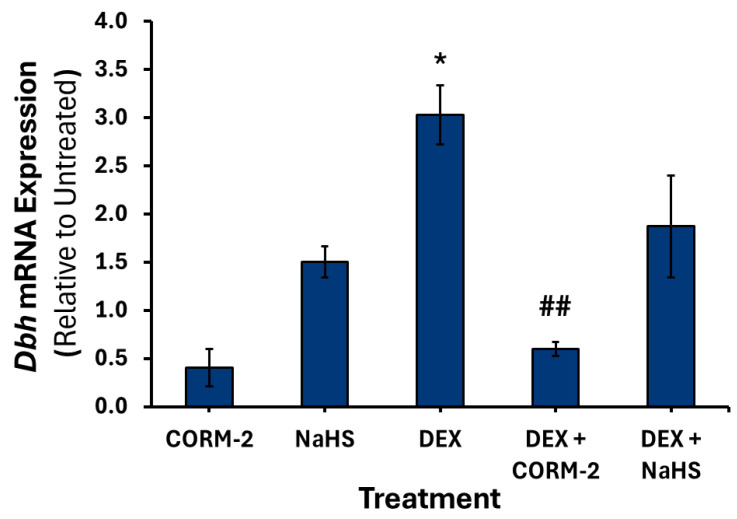
*Dbh* mRNA expression following dexamethasone and gas donor treatments. PC12 cells were pretreated with either 100 μM CORM-2 or 300 μM NaHS for 15 min, followed by treatment with or without 1 μM DEX (*n* = 3) for 6 h. Data are presented as the average fold change ± SEM of *Dbh* expression normalized to *Gapdh* and *Rpl32* and relative to an untreated control across three replicates (*p < 0.05, ## *p* < 0.01). * Indicates a significant difference from untreated control, # represents a significant difference from DEX treatment.

**Figure 10:**
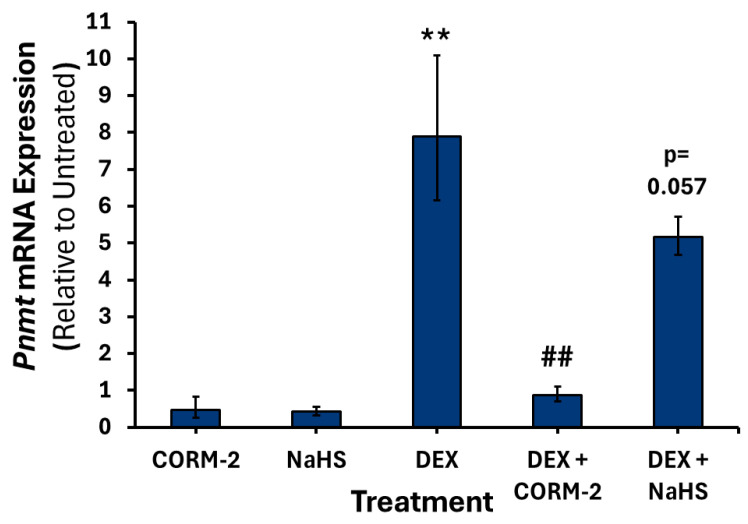
*Pnmt* mRNA expression following dexamethasone and gas donor treatments. PC12 cells were pretreated with either 100 μM CORM-2 or 300 μM NaHS for 15 min, followed by treatment with or without 1 μM DEX (*n* = 3) for 6 h. Data are presented as the average fold change ±SEM of *Pnmt* intronless expression normalized to *Gapdh* and *Rpl32* and relative to an untreated control across three replicates (**/## *p* < 0.01). * Indicates a significant difference from untreated control, # represents a significant difference from DEX treatment.

Treatment with DEX alone increased the expression of *Th* 1.7-fold (*p* < 0.05) (Figure 8), *Dbh* 3-fold (*p* < 0.05) (Figure 9), and *Pnmt* intronless 7.8-fold (*p* < 0.01) (Figure 10). Pretreatment with 100 μM CORM-2 significantly attenuated the DEX-mediated increase in *Th* (*p* < 0.001), *Dbh* (*p* < 0.01) and *Pnmt* (*p* < 0.01) mRNA expression. In contrast, pretreatment with 300 μM NaHS did not significantly alter DEX-mediated increase in any of the catecholamine biosynthesis genes. Notably, both DEX-treated mRNA levels of *Dbh* and *Pnmt* intronless were not significantly different from those of untreated controls after NaHS pretreatment.

### 3.5. Effects of CO and H_2_S on the DEX-Mediated Catecholamine Content in Culture Media

**Figure 11:**
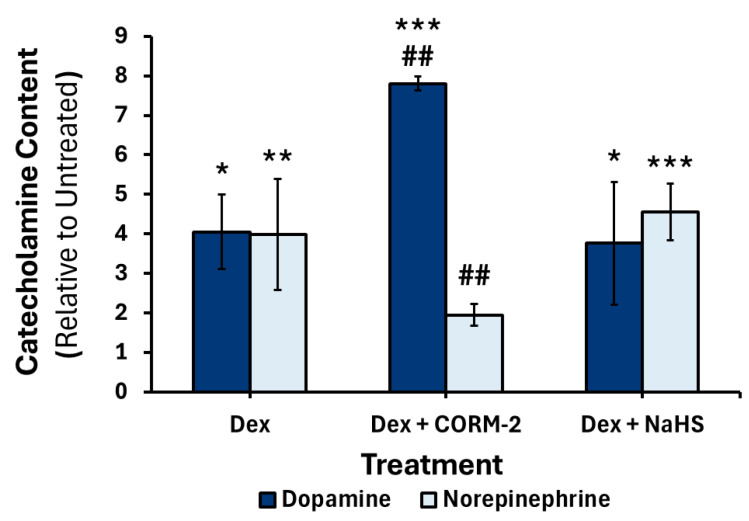
Changes in the 24 h glucocorticoid-mediated catecholamine content in culture media of dopamine and norepinephrine in response to CORM-2 or NaHS pretreatment. PC12 cells were pretreated with either 100 μM CORM-2 or 300 μM NaHS, followed by a DEX treatment for 24 h. Data are presented as the average concentration ±SEM of dopamine (*n* = 2) or norepinephrine (*n* = 3) relative to untreated controls (* *p* < 0.05, **/## *p* < 0.01, *** *p* < 0.001). * Indicates a significant difference from untreated control; # represents a significant difference from DEX treatment.

There was no change in catecholamine content after 6 h (data not shown). After 24 h, DEX treatment significantly increased norepinephrine levels 3.9-fold (*p* < 0.01) (Figure 11); this increase was attenuated by pretreatment of CORM-2 (*p* < 0.01) but not NaHS, which showed a 4.5-fold (*p* < 0.001) increase compared to untreated control (Figure 11). After 24 h, DEX treatment significantly increased dopamine levels 4.0-fold (*p* < 0.01); this response was potentiated by pretreatment with CORM-2 (*p* < 0.01), resulting in a 7.7-fold (*p* < 0.001) increase in dopamine levels, compared to untreated control (Figure 11). Pretreatment with NaHS did not significantly affect the DEX-mediated increase in dopamine content (Figure 11).

## 4. Discussion

This study aimed to investigate the role of CO and H_2_S in regulating catecholamine production and release. In the present study, exogenous CO donation influenced the expression of enzymes involved in catecholamine biosynthesis. The doses of CORM-2 used in this study are below levels that induce cytotoxic effects in PC12 cells, with doses of 400 μM or higher for 6 h inducing cell death [36], suggesting that the resultant changes in catecholamine gene expression and content in media are dependent on the signaling action of CO. At the same time, there was no change in the expression of catecholamine biosynthesis genes following treatment of cells with the H_2_S donor NaHS. While H_2_S has been shown to induce the transient release of catecholamines [33,34], it is possible that the single NaHS treatment only induced a small amount of catecholamine at the start of our treatment, which would be insignificant over 24 h of basal release. NaHS releases H_2_S rapidly when in solution, with a half-life of about 5 min [37]. Therefore, the use of a single donation of NaHS limits the relevance of this study to the effects of exogenous donation of high concentrations on catecholamine biosynthesis. To determine the definitive role of H_2_S in catecholamine biosynthesis, periodic treatment with NaHS, use of slow-release donors, or induction of basal H_2_S production should be carried out in future studies. Alternatively, the role of H_2_S may be limited to maintaining mitochondrial function and glucocorticoid production in the adrenal cortex [32]. Glucocorticoids are extremely important in regulating catecholamines, particularly the expression and activity of PNMT [6,38]. Therefore, it is possible that the effects of H_2_S may be driven solely by H_2_S in the adrenal cortex, and NaHS treatment in adrenal chromaffin cells, such as PC12, will not have any effect.

A significant but moderate decrease in *Th* and *Dbh* mRNA expression was observed in response to the higher CORM-2 concentrations of 50, 80, and 100 μM. Interestingly, the expression of *Pnmt* did not follow the same trend. Instead, there was a significant but moderate increase in *Pnmt* intronless mRNA expression after treatment with 20 and 100 μM of CORM-2. The difference in response is interesting, considering the similarities in the promoter regions of each gene, which contain many common elements and are bound by similar transcription factors [39]. There are, however, some notable differences between the *Th*, *Dbh* and the *Pnmt* promoters. One difference is in the mechanism of responsiveness to cyclic adenosine monophosphate (cAMP)/protein kinase A (PKA) signalling. Both *Th* and *Dbh* have cAMP response element (CRE) motifs that have been capable of interacting with CRE-binding proteins (CREBs) [40,41], while the binding of early growth factor (Egr1) drives the cAMP responsiveness in the *Pnmt* promoter [42]. However, no direct evidence exists of exogenous CO donation inducing a cAMP response. Future studies are required to determine if this difference is due to a differential cAMP response.

Interestingly, both the *Th* and *Dbh* promoters contain activating protein-1 (AP-1) and homeodomain core recognition (HD) sites that enhance or participate in promoter response to cAMP [41]. These HD sites are binding sites for paired-like homeobox (Phox2, also known as Arix) proteins [43]. Phox2 proteins have been implicated in nervous system development, particularly in noradrenergic phenotypes [44]. The CRE element in the *Dbh* promoter overlaps with several other motifs, including AP-1 and an HD site [43]. *Dbh* requires interactions between Phox2, AP-1 proteins and CREB-binding proteins (CBP) for maximal response to cAMP [43]. After cAMP treatment, the AP-1 proteins bind to the CRE/AP-1/HD element switch from c-Jun and JunD to c-Fos, c-Jun, and JunD [41]. Similarly, the *Th* promoter also contains homeodomain core recognition (HD) sites proximal to an AP-1 motif, although these do not overlap with its CRE [41,45]. The binding of Phox2 to the HD site depends on the proximal AP-1 motif [41] but does not act synergistically with CREB [46]. These related AP-1/Phox2 mechanisms in the *Th* and *Dbh* promoters may explain the differential responses observed in our CORM-2 treatments.

PC12 cells were initially described as a noradrenergic cell line [47] before the discovery of low levels of *Pnmt* mRNA expression. To that end, Phox2a and Phox2b proteins have been observed in PC12 cells [48], and non-noradrenergic cell lines require transfection with Phox2 expression plasmids to exhibit increases in DBH activity in response to cAMP [46]. While there is no evidence of CO’s involvement in Phox2 production and activity, CO has been shown to regulate AP-1 proteins by modulating mitogen-activated protein kinase (MAPK) pathways [49]. CO has been implicated in extracellular signal-regulated kinase (ERK), c-Jun N-terminal kinase (JNK) and p38 MAPK pathways, often in an antiapoptotic role [49]. CORM-2 treatment has been shown to attenuate interleukin-1 beta (IL-1β)-induced activation of MAPK and AP-1 binding activities [50]. In PC12 cells, CO ameliorated nitrosative stress through MAPK pathways [51]. We hypothesize that the decrease in *Th* and *Dbh* mRNA expression is driven by CORM-2 inactivation of MAPK-mediated AP-1 protein-binding activities, and possibly the sequential inability of Phox2 to bind and lower transcription. Furthermore, the proposed mechanism would not affect the *Pnmt* promoter, which contains no known AP-1 or HD sites. The small G protein Rap1, a component of ERK MAPK signalling, has been identified as a contributor to cAMP/PKA upregulation of *Dbh* expression [41]. NO has been shown to inhibit Rap1 [52] and is a promising target for CO-dependent ERK-MAPK-pathway inhibition. To determine the differential effects of CO on *Th, Dbh* and *Pnmt* mRNA expression, future studies should investigate changes in transcription factor mRNA and protein expression after CORM-2 treatment, specifically AP-1 proteins and Phox2. Catecholamine biosynthesis gene promoter activity should also be examined. Additionally, the activation and inhibition of MAPK pathways can elucidate these differential effects, particularly confirming whether CO affects Rap1 activity.

The response of *Th*, *Dbh* and *Pnmt* to CO differed drastically from previous work with NO, in which exogenous donation of NO to PC12 cells by treatment with 100 μM SNP for 6 h resulted in significantly increased mRNA expression of *Th* 2.0-fold, *Dbh* 1.3-fold, and *Pnmt* intronless 2.0-fold [28]. This suggests a differential response of catecholamine biosynthesis genes to CO and NO. The CO and NO signalling pathways have similar targets and are involved in crosstalk [53]. CO and NO have been shown to activate soluble guanylate cyclase (sGC), the enzyme that catalyzes cyclic guanosine monophosphate (cGMP) production [53]. Treatment of PC12 cells with SNP increases cGMP content [54], and the upregulation of catecholamine biosynthetic genes by SNP in both PC12 and primary bovine adrenal medullary cells is dependent in part on the cGMP/cGMP-dependent protein kinase (PKG) pathway [28,55].

It is, therefore, interesting that CORM-2 treatment did not induce a similar cGMP-dependent increase in catecholamine biosynthesis gene expression. CO induction of cGMP and downstream signalling is less understood than that of NO and is a contentious area of research [56]. CO has a much lower affinity for and activation level of sGC compared to NO, resulting in only a 4–6-fold increase in activity, compared to the approximately 400-fold increase by NO [57]. Despite this difference in affinity, direct relationships between CO and cGMP have been confirmed in multiple systems [53]. One theory that reconciles these two facts is the potential of a molecule that sensitizes sGC to CO. The benzyl indazole derivative YC-1, a known activator of sGC, also increases sGC activity in response to CO [57]. YC-1 stabilization of the active configuration of sGC increases CO-dependent stimulation of sGC to levels similar to those of NO [57]. However, since this discovery, no naturally occurring YC-1-like molecule has been identified [56].

Some evidence suggests that CO may stimulate the activity of sGC and increase cGMP levels in PC12 cells. When PC12 cells are exposed to nerve growth factor (NGF), they extend neurites, and this neurite growth can be further improved by guanosine treatment [58]. Treatment with sGC inhibitors abolishes guanosine’s enhancement of NGF-dependent neurite outgrowth [59]. It was also found that inhibiting HOs but not NOS affects guanosine-enhanced sGC-mediated neurite outgrowth [59]. Additionally, the combined treatment of NGF and guanosine activated HO-2 and induced the expression of HO-1 [59]. Inhibition of HOs led to a reduction in cGMP levels [59]. However, the role of CO in sGC-mediated neurite outgrowth was not independently verified by treatment with a CO donor.

We theorize three possible explanations for why exogenous CO did not increase the expression of catecholamine biosynthetic genes. (1) CO alone does not induce sGC activity in PC12 cells. It is possible that PC12 does not produce an unknown YC-1-like or other molecule required for high-affinity binding and complete activation. Alternatively, treatment with NGF, as performed previously [57], which has been shown to increase cGMP levels [58], may be required for CO to induce sGC activity in PC12 cells. (2) Our CORM-2 treatments did not induce enough sGC activity to increase gene expression. As CO has a lower affinity and a more negligible effect on sGC activity, it is possible that a 6 h treatment with a quick-release donor, such as CORM-2, was not sufficient to induce cGMP/PKG-dependent gene expression. (3) Activation of additional signalling pathways is required to induce catecholamine biosynthetic gene expression. Our previous work with NO revealed that inhibitors for PKA, PKC, cGMP, and PKG all independently attenuated SNP’s induction of catecholamine biosynthesis genes [28]. To determine the differential effects of CO and NO on catecholamine biosynthesis genes, future studies should investigate changes in sGC activity and cGMP concentration after CORM-2 and SNP treatment, in combination, and at longer time points. The activation and inhibition of PKA, protein kinase C (PKC), and PKG pathways can also elucidate these differential effects.

Glucocorticoids significantly influence catecholamine biosynthesis, as the promoter regions of *Th*, *Dbh*, and *Pnmt* contain glucocorticoid response elements (GREs) [60,61,62]. Although the binding of glucocorticoid receptors (GRs) to the GREs within the *Dbh* promoter has not been conclusively confirmed, *Dbh* expression does respond to DEX [60]. Consequently, any alterations to the glucocorticoid response are likely linked to the modulation of the GR. CO has been shown to interact with GR. The HO-1 promoter contains a glucocorticoid response element; however, the direction of this response appears to be dependent on the cell type [63]. Therefore, it is plausible that exogenous CO donation attenuated DEX-mediated GR activity in PC12 cells, ameliorating the transcriptional response.

In the absence of glucocorticoids, GR resides in the cytosol in complex with several chaperones, including heat shock protein 90 (Hsp90) and heat shock protein 70 (Hsp70) [64]. These chaperone proteins prevent the degradation of GR and promote proper maturation of the protein, and coordination with Hsp90 and Hsp70 is required for GR’s high-affinity towards glucocorticoids [64]. Inhibition of Hsp90 results in GR degradation [65]. In human breast cancer cells, CO has been shown to inhibit Hsp90 activity and decrease the expression of some Hsp90 client proteins [66]. It is possible that CO’s ability to attenuate the DEX-mediated increase in mRNA expression of *Th*, *Dbh*, and *Pnmt* intronless may occur through modulation of Hsp90 activity, resulting in either increased GR degradation or a decrease in GR affinity towards DEX. However, in astrocytes, exogenous CO has been shown to stabilize hypoxia-inducible transcription factor-1α (HIF-1α) by increasing HIF-1α/Hsp90 interactions [67]. This suggests that CO modulation of Hsp90 activity plays different roles depending on the cell type or client protein. The CO-induced stabilization of HIF-1α by Hsp90 is also of interest, as HIF-1α is a critical transcription factor in the response of catecholamine biosynthesis genes to hypoxic conditions [68]. Additionally, HIF-2α is also vital to the development of catecholaminergic sympathoadrenal cells [69,70], and the basal production of epinephrine by regulating *Pnmt* [71]. This highlights the importance of HIFs in catecholamine biosynthesis. Determining how Hsp90 activity changes in PC12 cells after CO donation would help confirm its role in glucocorticoid and HIF-mediated regulation.

Cytochrome P450 3A4 (CYP3A4) is an enzyme that inactivates several pharmacological substances, including DEX [72,73]. Interestingly, CYP3A4 is inhibited by both CO and NO [74,75]. However, while active CYP3A4 has been identified in adrenocortical carcinomas [76], it is unclear whether adrenal medullary or PC12 cells express this isoform. Given that CO ameliorated the DEX response of all three catecholamine biosynthesis genes, it seems unlikely that CO-dependent inhibition of CYP3A4 occurred. It is also important to note that a previous study utilized a photo-activated CORM to inhibit CYP3A4, and it is, therefore, possible that the difference in release rate may contribute to the differential response [75]. It is also possible that NO, which potentiates the DEX response in *Th*, *Dbh*, and *Pnmt* [28], may inhibit CYP3A4 or a homologous protein in PC12 cells, while CO is unable. Investigating CYP3A4 or other cytochrome P450 enzymes may elucidate the differential response of CO and NO to DEX-mediated upregulation of catecholamine biosynthesis genes.

Circadian rhythm signalling is another pathway that may mediate the interactions between glucocorticoids, blood pressure, catecholamines and carbon monoxide. Whole transcriptome analysis of DEX programmed Wistar–Kyoto rats and SHR has highlighted circadian signalling as a potential mechanism of the development of hypertension [77]. Both dopamine degradation, as catalyzed by monoamine oxidase, and dopamine synthesis, as catalyzed by TH, are under clock influence [78]. In particular, the circadian rhythm factor Neuronal PAS domain protein 2 (NPAS2) expression was decreased in SHR [77]. NPAS2 is a CO-responsive factor [79,80]. NPAS2 contains a heme motif, which controls its transcriptional activity, with CO binding inhibiting DNA binding [80]. Future studies should investigate the transcriptional regulation of *Th*, *Dbh* and *Pnmt* by NPAS2 as a potential key modulator of catecholamine biosynthesis through circadian signalling, glucocorticoids and CO.

The DEX-mediated increase in norepinephrine levels was attenuated when cells were pretreated with CORM-2. This result aligns with the similar attenuation of the DEX-mediated increase in the mRNA expression of *Dbh*, suggesting the decrease likely affects the levels of translated DBH. However, this study did not measure DBH protein levels and enzyme activity, and CO may be affecting the production of DBH at steps other than transcription. Future studies should investigate the catecholamine biosynthetic enzyme levels and activity. Interestingly, there was an increase in the amount of dopamine in the cell culture media after treatment with CORM-2 and DEX compared to DEX alone. This is in contrast to the significant attenuation of the DEX-mediated increase in the mRNA expression of TH, the rate-limiting enzyme responsible for dopamine production [81]. This suggests that CO may be affecting protein translation, stability, activity, or dopamine release in addition to affecting gene transcription. CO has been shown to induce dopamine release [82]. Rats exposed to pure CO gas for short intervals showed significantly higher dopamine concentrations in the striatum [83]. Additionally, pure CO gas reduces dopamine reuptake [84]. While these studies use vastly higher concentrations of CO than those used in this study, they show a link between CO and dopamine release. Exposure of neuronal stem cells to lower (~25 ppm) intermittent exposures of CO using a novel CORM resulted in an increase in cell survival and dopaminergic differentiation [85]. Additionally, the CO exposure increased *Th* mRNA and protein expression [85]. A treatment using 100 μM CORM-2 in PC12 cells decreased *Th* mRNA expression. The discrepancy between results may be due to differences in cell type, dosage, or dosage timing and duration. CORMs with different chemistry and release rates have been shown to modulate different pathways [86]. Regardless, these results highlight the ability of CO to modulate dopamine production and release. It is important to highlight the preliminary nature of this study’s analysis of catecholamine content in media. Future studies should investigate changes in catecholamine content at longer time points, repeated gas donation, and with additional replicates to ensure statistical reliability. Additionally, the use of liquid chromatography–mass spectrometry would allow for high-sensitivity detection of catecholamines.

TH differs from DBH as, in addition to being transcriptionally activated by DEX, it is also enzymatically activated by DEX [87,88]. This activation may be a result of phosphorylation of Ser31 [89], a critical serine residue [90]. Glucocorticoids, including DEX, have been shown to activate the catalytic and regulatory activities of the cAMP/PKA pathway; however, the exact mechanism remains unclear [91,92]. Additionally, 100 μM of CORM-2 has been shown to inhibit cAMP production induced by forskolin in bronchial epithelial cells [93], and increase cAMP levels in serum-starved pluripotent stem cells [94]. Therefore, it is plausible that despite CO inhibiting *Th* gene expression through GR-dependent means, DEX and CO are increasing TH enzymatic activity through GR-independent means, namely phosphorylation of critical serine residues on TH via activation of the cAMP/PKA pathway. This could explain why CO potentiated the production and release of dopamine despite ameliorating the DEX response on the transcription of *Th*. In the present study, changes in the protein levels of catecholamine-synthesizing enzymes were not analyzed; future studies should investigate the effects of gas transmitters on catecholamine biosynthesis enzyme expression.

## 5. Conclusions

In summary, this study demonstrates that exogenous CO, but not H_2_S, regulates catecholamine biosynthetic enzymes. CO decreased *Th* and *Dbh* expression while slightly increasing Pnmt and attenuated the DEX-mediated upregulation of these genes, consistent with possible effects on glucocorticoid signalling. Short-term H_2_S treatment did not alter catecholamine biosynthetic gene expression or catecholamine content, suggesting its role may be limited or indirect. CO also produced differential effects on dopamine and norepinephrine release, underscoring its distinct actions. Taken together, these findings identify CO as a novel regulator of catecholamine biosynthesis and highlight essential differences in how gas transmitters influence stress-related pathways.

## Figures and Tables

**Table 1:** Primers Utilized for qPCR.

Gene (Accession Number)		Sequence (5′–3′)	Annealing Temperature (°C)
*Gapdh* (NM_017008.4)	Forward	GTCATCCCAGAGCTGAACGG	60
	Reverse	ATACTTGGCAGGTTTCTCCAGG	
*Rpl32* (NM_013226.3)	Forward	GGTGGCTGCCATCTGTTTTG	60
	Reverse	GTTTCCGCCAGTTTCGCTTAAT	
*Th* (NM_012740.4)	Forward	GCGACAGAGTCTCATCGAGGAT	58
	Reverse	AAGAGCAGGTTGAGAACAGCATT	
*Dbh* (NM_013158.3)	Forward	CGGTTTCTCCGACTGGAAGT	60
	Reverse	ATCAAGGGCGTGTACACCAG	
*Pnmt* Intronless (NM_031526.2)	Forward	CGAGGACAAGGGAGAGTCCT	60
	Reverse	GGGCTTGTGCACATCAATGG	

## Data Availability

The original contributions presented in this study are included in the article. Data inquiries can be directed to the corresponding author. PC12 cells were obtained from the University of California, San Diego (UCSD, San Diego, CA, USA).

## Abbreviations

The following abbreviations are used in this manuscript:

3MST	3-Mercaptopyruvate Sulfurtransferase
AP-1	Activating protein-1
CRE	cAMP response element
CO	Carbon monoxide
CBS	Cystathionine β-synthase
CSE	Cystathionine γ-lyase
CYP3A4	Cytochrome P450 3A4
DEX	Dexamethasone
DBH	Dopamine β-hydroxylase
Egr1	Early growth factor
ELISA	Enzyme-linked immunosorbent assay
ERK	Extracellular signal-regulated kinase
GR	Glucocorticoid receptor
GRE	Glucocorticoid response element
GCs	Glucocorticoids
GAPDH	Glyceraldehyde 3-phosphate dehydrogenase
Hsp70	Heat shock protein 70
Hsp90	Heat shock protein 90
HO	Heme oxygenase
HD	Homeodomain core recognition
H_2_S	Hydrogen sulfide
HPA	Hypothalamic–pituitary–adrenal
HIF-1α	Hypoxia-inducible transcription factor-1α
IL-1β	Interleukin-1 beta
mRNA	Messenger RNA
MAPK	Mitogen-activated protein kinase
NGF	Nerve growth factor
NO	Nitric oxide
NPAS2	Neuronal PAS domain protein 2
PNMT	Phenylethanolamine N-methyltransferase
PKA	Protein kinase A
PKC	Protein kinase C
RT-qPCR	Real-time quantitative polymerase chain reaction
Rpl32	Ribosomal Protein L32
NaHS	Sodium hydrosulfide
SNP	Sodium nitroprusside
sGC	Soluble guanylyl cyclase
SHR	Spontaneously hypertensive rat
TH	Tyrosine 3-hydroxylase
